# The importance of a “socially responsible” approach during COVID-19: the invisible heroes of science in Italy

**DOI:** 10.1186/s13054-020-02998-0

**Published:** 2020-05-26

**Authors:** Filippo Sanfilippo, Elena Bignami, Ferdinando Luca Lorini, Marinella Astuto

**Affiliations:** 1 Department of Anesthesiology and Intensive Care, AOU Policlinico-Vittorio Emanuele, University of Catania, Catania, Italy; 2 Anesthesiology, Critical Care and Pain Medicine Division, Department of Medicine and Surgery, University of Parma, Parma, Italy; 3 Emergency and Intensive Care Department, ASST Papa Giovanni XXIII, Bergamo, Italy

Dear Editor,

We would like to emphasize the importance of “socially responsible” approaches from physicians and societies during the coronavirus disease-2019 (COVID-19) pandemic. In Italy, a valuable example has been provided by the intensive care (ICU) community. It is important that other disciplines follow such “socially responsible” behavior, as irresponsible communication has generated potentially dangerous consequences. We summarize the “socially responsible” approach of our ICU community in three key points.

Cornerstone has been the avoidance of “public notoriety” at all costs, even when journalists are eager to obtain “shocking” news and notoriety is easily gained. “Shocking” news increase audience, sales, and sharing, but diffusion of unsupported information irresponsibly generates public disorientation with loss of guidance. Ironically, ICU physicians commented we desperately need football games back, so that millions of people become again football managers rather than COVID-19 experts!

The second key point is the identification of few strategic scientists delivering information. Only Crisis-Unit Coordinator for Lombardy ICUs and a couple of highly respected scientists who have (or held) apical positions at national and/or international level (SIAARTI and ESICM) were in charge to talk. One of them was recognized as “Healthcare Hero” [1]. Few other experienced physicians (ICU Director in “red areas” of North Italy) fighting COVID-19 on the frontline rarely appeared on TV programs for short communications.

The third key point is the scientific interaction between ICU physicians. They preferred to interact scientifically via webinars with the idea of sharing knowledge gained on the battlefield, generating protocols and, hopefully, improving outcomes of COVID-19. Such webinars were responsibly promoted by both SIAARTI and ESICM.

In summary, Italian ICU physicians avoided “compulsory public notoriety,” behaving as “invisible heroes of science.”

Unfortunately, the same has not happened in other disciplines with compulsory appearance on TV, social media, and newspapers by physicians with low *h-index*, predatory publication attitude, and no experience in coronavirus delivering highly misleading and scientifically unsupported information. Among other “self-proclaimed experts,” we had previous “Nobel-prize candidates” stating the coronavirus will disappear in summer or others reporting 100% survival on a couple of patients treated with a drug, generating false beliefs in the population. Such approach is “socially irresponsible” and should be stopped.

More than ever, laypeople should be maturely informed. A “socially responsible” approach to public information should be implemented to all fields involved in COVID-19, and the one delivered by the Italian ICU “invisible heroes” should be a leading worldwide example for other disciplines and countries.

## Data Availability

Not applicable

## Abbreviations

ESICM: European Society of Intensive Care Medicine
ICU: Intensive care unit
SIAARTI: *Società Italiana di Anestesia Analgesia Rianimazione e Terapia Intensiva*
